# Assessing early childhood developmental functioning in the parental screening tool: an application of the Rasch model

**DOI:** 10.3389/fpsyg.2025.1441174

**Published:** 2025-05-20

**Authors:** Lucia Ráczová, Tomáš Urbánek, Erika Jurišová, Marta Popelková, Tomáš Sollár

**Affiliations:** ^1^Department of Psychological Sciences, Faculty of Social Sciences and Health Care, Constantine The Philosopher University in Nitra, Nitra, Slovakia; ^2^Institute of Applied Psychology, Faculty of Social Sciences and Health Care, Constantine The Philosopher University in Nitra, Nitra, Slovakia

**Keywords:** developmental functioning, S-PMV11, Rasch model, Guttman scaling, developmental difficulties, parental screening tool, early childhood

## Abstract

**Objectives:**

Ensuring rapid and efficient detection of developmental difficulties in early childhood necessitates aligning screening tools with the timing of preventive examinations in each country, emphasizing the need for quick and effective unidimensional screening methods.

**Aim:**

This study aims to assess the scalability and unidimensionality of developmental functioning in two- and three-year-old children using Guttman scaling and the Rasch model.

**Methods:**

The anonymized data from 1,640 children aged 26 to 44 months, whose caregivers completed the S-PMV11 method, were gathered from routine pediatric preventive check-ups via the National Database.

**Results:**

The findings indicate the effective scalability of developmental functioning using the Guttman approach, thus enabling the early identification of children at risk. Additionally, the Rasch model confirms the unidimensionality of developmental functioning, highlighting the importance of early intervention addressing.

**Conclusion:**

Despite the instrument’s construct validity, significant concerns arise regarding its ability to capture children with borderline developmental functioning. These concerns could be addressed and improved by simply organizing the items by difficulty level in the pediatric response sheet, allowing the pediatrician to effectively identify any early signs of developmental issues.

## 1 Introduction

Developmental difficulties and neurodevelopmental disorders identified in the early years of life are significantly less numerous than their actual prevalence, not only in the at-risk population but also in the general population of children (Olusanya et al., 2023; Boyle et al., 2011). It is estimated that only about 30% of children with these difficulties are identified before entering school (Palfrey et al., 1987). However, recent systematic review (e.g., Francés et al., 2022) report much wider variability in prevalence rates of developmental difficulties and neurodevelopmental disorders, fluctuating globally between 4.70 and 88.50%, influenced by methodological differences, sociocultural factors, and disparities in diagnostic practices and professional training. As a result, early detection programs are increasingly recommended to recognize early warning signs of developmental difficulties and to inform the development of policies aimed at supporting the most vulnerable segments of the population (Francés et al., 2023; Cibralic et al., 2023). The call for early identification of children at risk for developmental difficulties has resulted in the use of psychomotor development screening scales during pediatric preventive check-ups (Yunilda et al., 2023; Prieto et al., 2022; Lipkin et al., 2020; Oberklaid and Efron, 2005).

Developmental difficulty in children manifests through delays in reaching developmental milestones or atypical, limited expression of abilities and skills typical of the child’s age (Institute of Medicine, 2001). Developmental milestones reflect a child’s optimal developmental functioning and are manifested in the child’s skills engaged in everyday interactions with the environment (Misirliyan et al. 2023). Developmental functioning can be defined as an umbrella term for optimal psychomotor development and daily functioning of the child, thus representing a counterpart to disability and impairment. It is understood as the capacity for adequate interaction between an individual’s bodily functions, activities, and participation in environmental conditions (World Health Organization, 2007). The inadequate developmental functioning of the child is thought to elicit increased attention from pediatricians and early childhood development specialists (World Health Organization, 2007; Coelho and Pinto, 2018).

Widespread use of parental screening tools can capture child manifestations that may not be observed by the pediatrician during an examination in the clinic (Cibralic et al., 2023; Vitrikas et al., 2017). Parental screenings are a recommended method to refine clinical assessment of a child’s developmental functioning (Glascoe, 2005), as it has been shown that up to 80% of caregivers correctly identify abnormalities in a child’s development at the age of 2 years (Chawarska et al., 2007). Parental screening tools are therefore inexpensive and effective means of identifying children with developmental difficulties across the population (Prieto et al., 2022). Methodologies such as the Parent’s Evaluation of Developmental Status (PEDS) and its complementary part Developmental Milestones (PEDS: DM) and Ages and Stages Questionnaires (ASQ) are recommended by the American Academy of Pediatrics. However, the schedule of preventive examinations during which the screening can be performed and interpreted varies from country to country. Therefore, it is crucial that the screening tool corresponds to the schedule of preventive examinations in a particular country (Cibralic et al., 2023). A mismatch between the dates when screening is required and the schedule of checkups may present a barrier to its use (Lipkin et al., 2020). To align with the pediatric health care system in Slovakia, the Method for Monitoring and Screening for Developmental Difficulties (S-PMV) was developed. This tool is designed to screen for psychomotor development during the preventive checkups in general pediatric care (©S-PMV, Prof. K. Matulay Endowment Fund (EF), 2016–2021; Ministry of Health, 2021). The compulsory use of S-PMV11 during the preventive examination at the age of 2 or 3 years allows professionals to catch developmental difficulties before the child enters kindergarten. The tool has already established its structural validity and underwent norm standardization in 2013. The current study aims to further examine its structural validity using new, practice-based data, providing an updated perspective based on more recent findings.

Developmental screening tools should be optimized to be fit-for-purpose and sensitive to capturing development as a whole or discrete developmental domains (van Buuren and Eekhout, 2023). In the context of the S-PMV11 instrument, developmental functionality is viewed as unidimensional. A unidimensional screening instrument can produce a scale based on a hierarchy of dichotomous items that progress from the easiest to the most challenging, or from the less to the most extremely delineated (Bond and Fox, 2007). A simple scale meeting Guttman’s requirements would allow the pediatrician to verify the accuracy of the parental assessment during the preventive exam by directly administering the selected item that most closely matches the child’s current level of ability. Since the S-PMV11 is used at 2 or 3 years of age, it can be assumed that items will be more challenging for two-year-olds than for older children. Analyses will therefore be conducted separately for the two age groups. The first aim is to investigate the scalability of developmental milestones for two- and three-year-olds in the S-PMV11 instrument, which implies the research question RQ1: *Is the scalability of developmental milestones for two- and three-year-olds possible when using Guttman scaling?*

The process of detecting errors in response patterns is also known as scalogram analysis and forms the basis of the Rasch model (Engelhard, 2008). The Rasch model, which is based on the item response theory approach, considers a scale to be a set of homogeneous items (Jelínek et al., 2007). The constructed model then displays the probability of an individual with a certain level of latent ability responding to a particular item on a uniform scale (Embretson and Reise, 2000). Thus, the use of the Rasch model can contribute to validating the accuracy and sensitivity of capturing developmental functioning. Analyses will again be conducted separately for the two age groups. Research question 2 therefore focuses on the use of the Rasch model, RQ2: *Can developmental functionality be considered unidimensional when using the Rasch model in two-year-olds and three-year-olds?*

## 2 Methods

### 2.1 Design and data collection

The research design is quantitative, cross-sectional, and oriented toward examining and validating psychometric indicators of the developmental functionality domain in S-PMV11. Data from S-PMV11 were sourced from the National Database of Selected Developmental Indicators (©S-PMV, Prof. K. Matulay Endowment Fund (EF), 2016–2021). The study sample included anonymized data from children whose primary caregivers completed the screening between April 1, 2021, and December 31, 2021. The entire sample (*N* = 1,640) consisted of children aged 26 to 44 months. The children were further divided into younger children (< 35 months) and older children (≥ 36 months), according to the age criteria outlined in the S-PMV11 Manual (©S-PMV, Prof. K. Matulay Endowment Fund (EF), 2016–2021).

### 2.2 Measurement and instruments

The method whose psychometric properties were validated is called the Developmental monitoring and screening method for the 11th check-up in primary care (shortly S-PMV11). S-PMV11 is one of 10 screening tools that form a single series designed for monitoring the development of psychomotor functions and screening for developmental difficulties based on parental assessment in primary pediatric care. S-PMV11 specifically targets the detection of potential developmental risks in children aged 2 to 3 years. It is obligatory used in pediatric practice in Slovakia. The data were collected online during the 11th preventive check-up in general care for children and adolescents carried out at 2 and 3 years of age (Ministry of Health, 2021). The method was completed by the child’s caregiver.

S-PMV11 is a standardized screening tool designed for the early identification of deviations from typical development and behavior in children aged 2 and 3 years. Deviations identified indicate potential developmental risks, affecting the child’s functional abilities. This method comprises two versions: one tailored for younger children (aged 26–35 months) and one for older children (aged 36–40 months). Both versions are identical, differing solely in the percentile bands for norms, borderline cases, and risks. In the screening record, the pediatrician classifies children into three categories based on their developmental score percentiles. Developmental functioning sum score consists of 20 items mentioned below. A score above the 26th percentile falls within the normative range, indicating no significant deviations from the population norm. Scores between the 11th and 25th percentiles are classified as borderline, suggesting mild deviations requiring increased monitoring, while scores below the 10th percentile indicate elevated risk, warranting further evaluation and possible specialized care. According to standard pediatric procedures (©S-PMV, Prof. K. Matulay Endowment Fund (EF), 2016–2021), younger children scoring 17 to 20 points fall within the normative range, those scoring 14 to 16 points are classified in the borderline range, and those scoring 13 points or lower fall into the risk category. For older children, scores of 18 to 20 points indicate the normative range, scores of 16 to 17 points correspond to the borderline range, and scores of 15 points or lower place a child in the risk category.

The developmental functioning contains 20 items with dichotomous “*yes*”-"*not yet*” responses, focusing on the areas of motor skills (4 items, example of item: “*M3 Can draw a circle.*”); social behavior [3 items, example of item: *“SS2 Shows interest in other children (watches what they are doing, approaches them, joins in play).”*]; cognition [3 items, example of item: *“K2 Can assemble a picture made of at least 3 pieces (puzzle).”*]; speech understanding (3 items, example of item: *“PR3 Can answer the question ‘How old are you?’ or show the number using fingers.”*); speech production (3 items, example of item: *“H1 Speaks in sentences of three or more words.”*); self-care (3 items, example item: *“SE2 Can eat independently using a spoon.”*), and kindergarten readiness (1 item: *“Z1 Do you think your child is mature and ready for kindergarten?”*). The maximum possible score for developmental functioning is 20 points. The items in the pediatric response sheet denote various aspects of developmental functioning: M for motor skills, SS for social behavior, K for cognition, PR for speech understanding, H for speaking, SE for self-care, and Z for kindergarten readiness. Within each category, items are labeled with abbreviations such as M1, M2, M3, and M4 for motor skills. However, these numerical labels do not indicate the difficulty level of the items; they are simply identifiers, with the numbers not reflecting an increasing level of difficulty.

### 2.3 Data analysis method

To verify the scalability of developmental functionality from a deterministic conception of the Guttman scale (Aim 1), the achieved values of the coefficient of reproducibility (coefficient of reproducibility = CR) were investigated. The coefficient takes values from 0 to 1, with values higher than CR = 0.9 indicating acceptable unidimensional use of the scale. Examination of the scalability of the developmental functioning items by analyzing Guttman response patterns was conducted in R, specifically in the guttman package, version 0.1.0 (Libura, 2024).

The premise of the validated Rasch model (Aim 2) was the latent continuous capacity for child developmental functioning. After logarithmizing the data, we determined the difficulty of items in the population of children using an item difficulty index (denoted as b). The fit of the data to the model was assessed with a discriminant value fixed at 1 (*α* = 1) with the variance freely estimated. The model was estimated using the maximum likelihood method. Parameters such as χ^2^, *p*-value, RMSEA, SRMR, TLI, and CFI were examined to evaluate the fit of the data to the model. Interpretation of the Rasch model is similar to confirmatory factor analysis, where, as noted by Markland (2007), the significance of χ^2^ should not be the sole criterion for determining data fit due to conservatism, particularly in large research sets. Therefore, we also utilized the comparative fit index (CFI) and the Tucker-Lewis index (TLI), which are expected to be 0.9 or higher, as well as the standardized root mean square residual (SRMR) and the root mean square error of approximation (RMSEA), which should be less than 0.08 (Bond and Fox, 2013). An item fit index was computed for the items, providing a direct comparison of modeled and observed frequencies of correct and incorrect responses (Orlando and Thissen, 2000). Standardized and unstandardized item infit and outfit indices were also added, with too low values indicating overfitting typical of Guttman response patterns, indicating possible local dependence of the items. High values of fit, in turn, indicate unpredictability of responses (underfit), which may be due to item weakness or guessing on the part of the respondent. Standardized infit and outfit values in the range of −2 to +2 are considered ideal (Bond and Fox, 2013). Empirical reliability values were also investigated. The item characteristic functions, item information functions, and screening information function with measurement error were graphed. R software (R Core Team, 2024) and the packages readr (Wickham et al., 2024), tidyverse (Wickham et al., 2019), mirt (Chalmers, 2012), ggplot2 (Wickham, 2016), psych (Revelle, 2024), cowplot (Wilke, 2024) were used.

## 3 Results

In this section, we aim to achieve two objectives: Aim 1 focuses on assessing the scalability of developmental milestones in children aged 2 and 3 years old using Guttman scaling. Aim 2 involves verifying the S-PMV11 instrument using the Rasch model, examining both the developmental milestones and the overall model of developmental functioning in younger and older children. Through these aims, we seek to deepen our understanding of developmental milestones and functioning in young children.

### 3.1 Sample characteristics

The mean age for the younger children was *M* = 30.51 months (*SD* = 2.78; Mdn = 31; *min* = 26, *max* = 35), and for the older children, it was *M* = 36.8 months (*SD* = 1.17; *Mdn* = 36; *min* = 36, *max* = 44). The basic information of the sample, such as gender and information about the person who completed screening are displayed in Table 1.

**Table 1:** Sample characteristics.

Sample characteristics	Younger children (*n* = 1,336)		Older children (*n* = 304)	
	n	%	n	%
Gender				
Girls	622	46.56	139	45.72
Boys	714	53.44	165	54.28
Screening completed by				
Mother	1,101	82.41	248	81.58
Father	46	3.44	10	3.29
Both parents	181	13.55	42	13.82
Another person	8	0.60	4	1.32

Another person = Another person refers to someone other than the parent who takes care of the child, such as a grandparent or another caregiver.

### 3.2 Internal consistency

In our research, good values of internal consistency of developmental functionality were found for younger children *α* = 0.84 and *ω* = 0.87 and for older children α = 0.86 and ω = 0.88.

### 3.3 Scalability of developmental milestones using Guttman scaling

After calculating the scalability of developmental functioning in younger children (*n* = 1,336), the unidimensionality of the S-PMV11 instrument can be highlighted. In the S-PMV11, item abbreviations reflect aspects of functioning that are measured, e.g., K1 means the focus of the item on cognition. The items in the text denote various content of developmental functioning: M for motor skills, SS for social behavior, K for cognition, PR for speech understanding, H for speaking, SE for self-care, and Z for Kindergarten readiness. Items were ranked in order of difficulty from easiest to most difficult as follows: M4, SS1, PR1, SS2, K1, SE2, M1, M2, SS3, SE1, K3, PR2, PR3, K2, M3, SE3, Z1, H2, H1, H3 (for the names of the items, see Table 2). For a total of 26,720 data (1,336 respondents x 20 items), a reproducibility coefficient index of CR = 0.90 was calculated, which corresponds to the established Gutmman scaling criterion reported as 0.90.

**Table 2:** Item scores for the Rasch model in younger children.

Item	b	SE(b)	SX^2^	RMSEA SX^2^	df	*p*	Infit	Infit t_u_	Outfit	Outfit t_v_
M4: connects the parts of the blocks	−6.318	0.312	10.659	0.035	4	0.031	0.583	−2.184	0.193	−3.845
SS1: shares joy	−5.933	0.268	15.727	0.024	9	0.073	0.636	−2.109	0.190	−3.626
PR1: fetches the ball when asked	−5.540	0.232	29.743	0.038	10	0.001	0.611	−2.642	0.083	−4.340
SS2: is interested in other children	−4.965	0.191	42.573	0.041	13	0.000	0.937	−0.444	1.384	1.279
K1: inserts shapes correctly	−4.817	0.183	15.450	0.012	13	0.280	0.927	−0.567	0.663	−1.337
SE2: eats by spoon	−4.683	0.175	17.778	0.017	13	0.166	0.807	−1.742	0.395	−3.091
M1: jumps forward with both feet	−4.046	0.147	14.945	0.014	12	0.244	0.898	−1.158	0.619	−2.386
M2: walks up the stairs	−3.917	0.143	52.172	0.050	12	0.000	1.042	0.518	1.051	0.355
SS3: plays with dolls	−3.871	0.141	18.821	0.021	12	0.093	0.822	−2.268	0.726	−1.779
SE1: dresses up	−3.503	0.130	44.251	0.045	12	0.000	0.928	−1.015	0.938	−0.417
K3: assigns colors	−2.969	0.117	20.298	0.023	12	0.062	0.929	−1.238	0.768	−2.443
PR2: completes the storyline	−2.841	0.115	26.816	0.030	12	0.008	0.807	−3.742	0.662	−4.014
PR3: answers about his/her age	−2.630	0.111	7.765	0.000	12	0.803	0.814	−3.885	0.610	−5.351
K2: composes a picture	−2.480	0.108	28.490	0.035	11	0.003	0.905	−2.005	0.749	−3.502
M3: draws a circle	−2.368	0.107	62.608	0.059	11	0.000	0.981	−0.404	0.860	−1.972
SE3: communicates a need in a timely manner	−2.136	0.103	9.952	0.000	11	0.535	0.801	−4.931	0.664	−5.886
Z1: is ready for Kindergarten	−2.109	0.103	10.381	0.000	11	0.496	0.758	−6.145	0.612	−7.064
H2: uses prepositions	−1.717	0.098	53.949	0.057	10	0.000	0.607	−12.071	0.488	−12.074
H1: says 3 or more words	−1.687	0.098	61.151	0.066	9	0.000	0.595	−12.611	0.476	−12.599
H3: uses the past tense	−1.576	0.097	48.102	0.057	9	0.000	0.628	−11.795	0.544	−11.023

b, difficulty; SE(b), standard error of estimate for difficulty; SX^2^, item fit index (Orlando and Thissen, 2000); RMSEA, root mean square error of approximation for SX^2^; df, degrees of freedom; *p*, significance of item fit (<0.05); infit/outfit, item fit; infit tu, standardized infit; outfit tv, standardized outfit; content of items: M, motor; SS, social behavior; K, cognition; PR, speech understanding; H, speaking; SE, selfcare; Z, Kindergarten readiness. The items from the S-PMV11 are presented in an abbreviated version.

For older children, the items were ranked in a different order of difficulty, with item Z1 being the most challenging. The order from easiest to most difficult item was as follows: SS1, SS2, M4, PR1, SE2, M1, SS3, K1, M2, PR3, K3, PR2, SE1, M3, K2, SE3, H1, H2, H3, Z1 (for the names of the items, see Table 2). For 6,080 data (304 respondents x 20 items), the index of coefficient of reproducibility was at CR = 0.90, thus the S-PMV11 instrument for 3-year-old children meets the requirements of a unidimensional scale.

### 3.4 Rasch model of developmental functioning

In the overall developmental functioning scores, younger children achieved mean values of *M* = 17.61, *SD* = 3.14, *Mdn* = 19, *skew* = −1.78, *kurt* = 3.41, *min* = 2, *max* = 20. Up to 529 younger children out of 1,336 achieved full scores, which corresponds to approximately 39.6%. For developmental functioning, older children scored *M* = 17.74, *SD* = 3.15, *Mdn* = 19, *skew* = −1.93, *kurt* = 3.87, *min* = 4, *max* = 20. For older children, 128 older children out of 304 achieved a full score, accounting for 42.11% of the responses. Together with the skewness and kurtosis indices, the descriptive statistics highlight the ceiling effect of the S-PMV11 instrument. According to the normative ranges defined in standard procedures for primary pediatrics, 74.0% (*n* = 984) of the younger children in the research sample fell within the normative range, 15.7% (*n* = 209) were in the borderline range, and 10.3% (*n* = 137) were in the risk range. Among the older children, 68.6% (*n* = 208) were classified within the normative range, 13.5% (*n* = 41) in the borderline range, and 17.8% (*n* = 54) in the risk range.

#### 3.4.1 Results of the Rasch model for younger children

**Figure 1:**
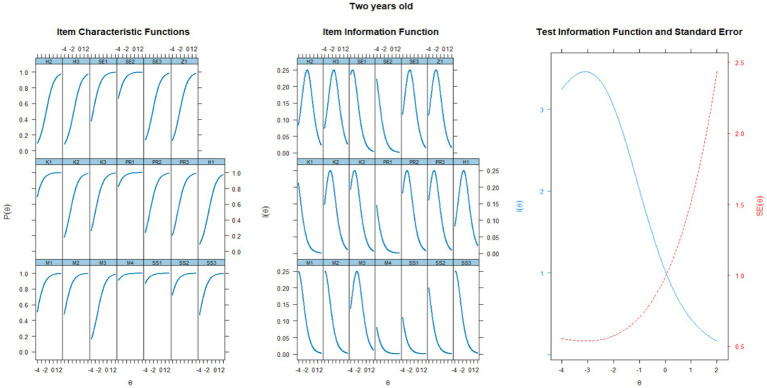
Characteristic curves of items, information functions of items, and test information function for a group of two-year-old children.

The fit of the younger children’s data (< 35 months) to the Rasch model is acceptable and shows indices: *χ^2^* = 1,309 (189, n = 1,336), *p* < 0.001; RMSEA = 0.067 [CI 95% = 0.063–0.070]; SRMSR = 0.098; TLI = 0.924; CFI = 0.924. The significance of chi-square statistics may be biased by the sample size, and therefore, when describing the results, we mainly focus on the RMSEA and TLI indices, which showed a satisfactory fit of the model to the data. The empirical reliability was at 0.730 and we consider it acceptable. The results for the items are shown in Table 2. The items showing the greatest variability in the SX^2^ indicator were the items focusing on drawing a circle (M3), walking up stairs (M2), and talking (H1, H2, and H3). While the item on joining the pieces of a building block (M4) is minimally challenging, the items focusing on speaking are among the most challenging indicators. Based on the parameter b, as well as looking at the item characteristic functions, item information functions, and test information function shown in Figure 1, it can be concluded that the items measure too low an estimated developmental functional ability. In the S-PMV11, the theta indicator provides the most information about ability at the −3 level, which corresponds to approximately three standard deviations from the norm.

#### 3.4.2 Results of the Rasch model for older children

**Figure 2:**
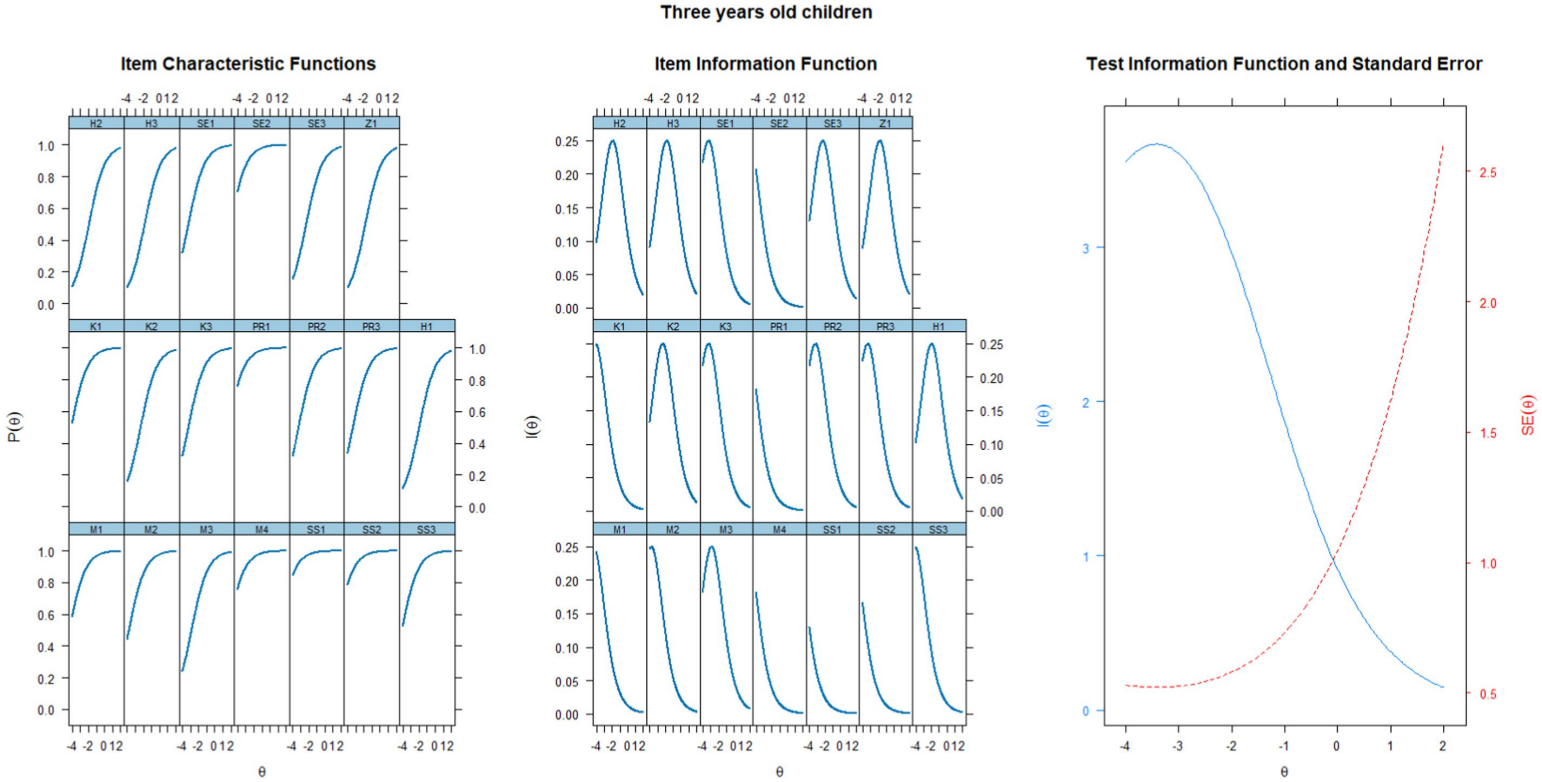
Characteristic curves of items, information functions of items, and test information function for a group of three-year-old children.

The data fit of older children (≥ 36 months) with the Rasch model is acceptable, with the following indices: χ^2^ = 464.225 (df = 189, *n* = 304), *p* < 0.001; RMSEA = 0.069 [CI 95% = 0.061–0.077]; SRMSR = 0.112; TLI = 0.924; CFI = 0.925. The RMSEA and TLI indices demonstrated satisfactory model fit. The empirical reliability was at the level of 0.721, which we consider acceptable. Results for individual items with abbreviated content are displayed in Table 3, and the visualization of curves is provided in Figure 2. Concerning the capture of age differences in the construct of developmental functionality, highlighting the SX^2^ indicators as well as to the infit and outfit indicators for speech items, which are less variable among older children compared to younger ones. While these captured differences provide evidence of the tool’s construct validity, we highlight the test information function, indicating that in older children, screening most accurately identifies children at a level of approximately −3.5 latent ability.

**Table 3:** Item scores for the Rasch model in older children.

Item	b	SE(b)	SX^2^	RMSEA SX^2^	df	*p*	Infit	Infit t_u_	Outfit	Outfit t_v_
SS1: shares joy	−5.703	0.507	NaN	NaN	0	NaN	0.801	−0.474	0.389	−0.746
SS2: is interested in other children	−5.312	0.443	0.743	0.000	1	0.389	0.908	−0.214	0.469	−0.749
M4: connects the parts of the blocks	−5.152	0.421	3.622	0.052	2	0.163	0.763	−0.824	0.186	−1.830
PR1: fetches the ball when asked	−5.152	0.421	5.021	0.071	2	0.081	0.666	−1.257	0.161	−1.957
SE2: eats by spoon	−4.879	0.387	2.183	0.017	2	0.336	0.772	−0.905	0.382	−1.314
M1: jumps forward with both feet	−4.360	0.335	10.376	0.049	6	0.110	1.051	0.314	0.829	−0.293
SS3: plays with dolls	−4.115	0.315	5.923	0.000	8	0.656	0.854	−0.760	0.465	−1.699
K1: inserts shapes correctly	−4.115	0.315	9.496	0.025	8	0.302	0.747	−1.430	0.380	−2.105
M2: walks up the stairs	−3.772	0.291	11.830	0.040	8	0.159	1.066	0.448	0.865	−0.353
PR3: answers about his/her age	−3.332	0.266	6.566	0.000	8	0.584	0.815	−1.380	0.562	−2.061
K3: assigns colors	−3.236	0.261	5.582	0.000	8	0.694	0.760	−1.913	0.564	−2.163
PR2: completes the storyline	−3.236	0.261	12.301	0.042	8	0.138	1.001	0.049	0.696	−1.393
SE1: dresses up	−3.236	0.261	6.322	0.000	8	0.611	0.964	−0.226	0.737	−1.171
M3: draws a circle	−2.851	0.244	9.841	0.028	8	0.276	0.829	−1.520	0.711	−1.656
K2: composes a picture	−2.325	0.225	3.557	0.000	7	0.829	0.778	−2.466	0.590	−3.412
SE3: communicates a need in a timely manner	−2.291	0.224	14.447	0.059	7	0.044	0.853	−1.589	0.799	−1.524
H1: says 3 or more words	−1.963	0.215	5.256	0.000	7	0.629	0.665	−4.472	0.526	−4.984
H2: uses prepositions	−1.906	0.214	12.685	0.052	7	0.080	0.597	−5.662	0.449	−6.236
H3: uses the past tense	−1.823	0.212	15.024	0.062	7	0.036	0.641	−5.085	0.510	−5.582
Z1: is ready for Kindergarten	−1.796	0.211	5.597	0.000	7	0.588	0.772	−3.074	0.673	−3.463

b, difficulty; SE(b), standard error of estimate for difficulty; SX^2^, item fit index (Orlando and Thissen, 2000); RMSEA, root mean square error of approximation for SX^2^; df, degrees of freedom; *p*, significance of item fit (<0.05); infit/outfit, item fit; infit tu, standardized infit; outfit tv, standardized outfit; content of items: M, motor; SS, social behavior; K, cognition; PR, speech understanding; H, speaking; SE, selfcare; Z, Kindergarten readiness. The items from the S-PMV11 are presented in an abbreviated version.

## 4 Discussion

The aim of this study was to investigate the scalability and unidimensionality of developmental milestones in 2 and 3 year old children by using Guttman scaling and also to verify the S-PMV11 instrument using the Rasch model, both for developmental milestones and for the whole model of developmental functioning. Such approaches offer a more relevant interpretation of achievement scores compared to classical test theory (Tesio et al., 2024). Although it is rather rare to obtain data that fit a Guttman formula (Abdi and Williams, 2010), in the case of the S-PMV11 instrument, it has been shown that developmental milestones can be scaled in terms of the Guttman approach, for both age groups of children. The data for both age groups were also shown to fit the Rasch model, however, the test information function achieved at approximately −3 latent ability is questionable. Our findings need to be put in the context of the practical application of screening at the population level in order to search for potential developmental difficulties. The practical purpose of the tool is to capture children at approximately −1 latent ability level of developmental functioning as stated in the tool manual (©S-PMV, Prof. K. Matulay Endowment Fund (EF), 2016–2021). Despite the identified unidimensionality and support for construct validity of S-PMV11, our findings yielded significant information about the very low level of measured ability at the −3 deviation level. We question the clinical application of screening in capturing children at possible borderline risk at around −1 ability level of developmental functioning. Parents of children should be educated early on about options for supporting developmental functioning, as late identification of difficulties does not allow professionals to develop further developmentally supportive interventions that are most effective the earlier they are initiated (Guthrie et al., 2023; Lipkin et al., 2020). We are concerned that the tool is not sensitive enough to capture children with borderline levels of developmental functional ability and therefore children may be systematically overlooked despite the need for additional population-wide care. Interpretation of our findings is therefore a syncretization of the psychometric as well as the practical evaluation of the instrument.

It is possible to find certain common features in psychomotor development that are particularly useful for clinical practice, as they lead to the rapid recognition of possible developmental difficulties. In their own research on Guttman errors in response patterns, the authors Blanchin et al. (2016) outlined the use of these errors for detecting subgroups of respondents and subgroups of items; in our interpretation, we will focus on the items measuring speaking aspect, which also proved to be the most challenging in Rasch’s model. Interpretation through Guttman scaling combined with the Rasch model allows us to gain theoretical as well as practical qualitative insight into the items. The interpretation of the Rasch model needs to take into account the clinical interpretation (Tesio et al., 2024) of whether the fit indices for the items match the theoretical and practical applications. Based on our findings, it can be concluded that the data in both younger and older children demonstrated fit parameters for the Rasch model that are consistent with the interpretation of a unidimensional construct with increasing developmental difficulty when it comes to speaking expressions.

The items measuring speaking aspect in our case were the most homogeneous in content, therefore they were the most challenging items with the highest error rates and can register children who, although meeting the easier milestones, do not have sufficiently developed speaking skills. Thus, the parent perceives the child as ready for kindergarten despite not completing the speech items, as they may believe that this is a natural variability in speech (Holm et al., 2023). This interpretation is also consistent with the Kindergarten readiness item, which is most difficult only in the group of older children, whereas in younger children it is located before the items measuring speaking aspect. Thus, thanks to the Guttman formula, as well as to the infit and outfit values of the items in the Rasch model, it is possible to identify a heterogeneous group of children called “late talkers,” who show less frequent verbal imitation and reduced lexical production (Zuccarini et al., 2023). The research on phonological skills requires further investigation due to the interindividual variability of this population, the significant prevalence of which has been pointed out by other authors (Zuccarini et al., 2023; Suttora et al., 2021; Desmarais et al., 2008). At the same time, talking can be understood as the result of the integration of social interaction, child talk, or cognition, but also as a manifestation of symbolic thinking, with the onset of egocentrism as indicators of this period (Heo et al., 2011). Despite being the most challenging milestones among the other items, their difficulty corresponded to the low level of developmental functioning ability in both groups of children. Therefore, non-performance of these items in a population of children should be a significant indicator of possible developmental difficulty.

For two-year-old children, the easiest milestone in the Guttman scaling M4 (Builds blocks, can connect the pieces of the building block) was found to be item SS1 (Shares joy) and SS2, which saturate the content of engaging in play with other children (Köngäs et al., 2022) and are the basis for the development of other indicators of children’s functionality. Failure to meet these developmental milestones may have clinically significant interpretive value because they are milestones that are manifest in socialization, in the formation of self-esteem, or in the child’s globally perceived well-being. The child’s social behavior and engagement in play with other children can be termed social visual engagement (SVE), which is manifested by learning to interact socially with others while observing other people, playing with others, and listening to the conversations of others. If a child’s establishment of these fundamental expressions is absent between the ages of 24 and 36 months, they may even be significant clinical indicators of potential autism spectrum disorders (Klin, 2023).

From a clinical perspective, the distribution of data across a large sample shows that the developmental function bands for both younger and older children align closely with those defined in standard primary pediatric guidelines. For children within the normative range (negative results), it is important to communicate item completion and offer guidance to promote motor, communication, and play skills. Phrases such as “no significant differences from same-age peers” or “the score is similar to other children of the same age” can indicate low risk. For children in the borderline or risk range (positive results), the focus should shift to addressing unmet items and supporting family competencies. Communication may include statements like “slight differences from peers” or “the score is lower than typical, but we will verify this in a follow-up.” Follow-up evaluations or specialist referrals may be necessary. As a screening tool, it is important to note that while many items may appear easy, failure to complete these easier items, even in children classified within the normative range, can serve as a significant indicator that the child is not developing typically. Beyond scores, the difficulty of the items a child fails to complete is critical for accurate assessment. Misclassification can occur if a child fails to complete easier items, which may indicate a risk of uneven development. The pediatric response sheet currently groups items by broad developmental areas, such as motor skills or social behaviors, which does not account for the varying levels of difficulty among them. It would be more beneficial to organize the items based on their difficulty level. This approach would allow clinicians to more effectively identify patterns in the child’s responses and recognize where they struggle the most. For example, a pediatrician would be able to see more clearly if a child is unable to perform the three most challenging tasks, as opposed to simply noting that the child struggles with some tasks without understanding the level of difficulty involved. Such a system would provide more insight into the child’s developmental needs and help guide decisions on further care or interventions. This would allow clinicians to more easily identify patterns in the child’s responses based on the complexity of the items, making it easier to determine if additional care or interventions are needed. Our research demonstrates that organizing the items by difficulty allows for a more nuanced and accurate assessment. The Rasch approach in our study confirms that the instrument captures significant developmental concerns at the population level. Therefore, we recommend that the tool be used with expert caution when items are unmet. To aid pediatricians, we suggest ranking items by difficulty in the scoring sheet, which would streamline result interpretation and improve alignment with population trends. Although all items in the screening tool are important as potential “red flags,” our focus is not solely on categorizing children as “normal” or “at risk.” Rather, we aim to emphasize the importance of understanding certain items that may be naturally easier for most children. When a child fails to complete these tasks, it can serve as an important indicator, even if the child’s overall score falls within the normal range. This is one of the key implications of our study — some items may be considered developmentally simple for the general population. We believe that it is crucial for pediatricians, particularly in the fast-paced clinical setting, to have the ability to effectively identify potential issues. This approach would allow for more nuanced decision-making, rather than automatically assuming that a child is within the normative range based on the total score. Individual analysis of a parent’s response pattern can help identify critical developmental milestones where interventions may be needed. Thanks to Rasch’s approach, we have shown that the instrument captures at the population level those children who already have significant developmental problems. However, it is equally important to identify children who may benefit from more thorough monitoring and a more comprehensive view of their overall development. This would also support raising parental awareness about the importance of completing all items listed in the tool, ensuring that no potential developmental concerns are overlooked.

### 4.1 Limits and recommendations for further research

We consider the most serious limitation of this study to be the lack of demographic and other descriptive information of children and caregivers, which may be important intervening covariates. These include, for example, the child’s previous health problems or previously diagnosed illnesses. On the caregiver’s side, information such as caregiver age and educational attainment was absent, which may be related to inadequate assessment of the child’s functioning in the S-PMV11. This limit is caused by the effect of the data collection managed at the national level with no possibility to change the dataset. Missing information is available to the pediatrician in direct contact with the caregiver and through the child’s medical record. Therefore, our interpreted findings are also set in the context of population-wide use of the instrument, with the conclusion to point out the possibility of systematically overlooked children with possible developmental difficulties at a level not so obvious as −3 latent ability. We believe that providing more detailed information about the parent and child in the Database could reveal additional associations with respect to other covariates such as parent education or child health status. There is an urgent need in creating and validating tools that are used to capture children with potential developmental difficulties early in life. Other screening methods, such as the Ages and Stages Questionnaire (Squires and Bricker, 2009), use three response options based on whether the child is performing the activity regularly, sometimes, or not yet. The instructions indicate that parents may choose not to answer if they are unsure about the response. While it would be possible to verify this procedure in the context of the screening, the current version of the screening tool follows the guidelines outlined in the manual (©S-PMV, Prof. K. Matulay Endowment Fund (EF), 2016–2021), which advises parents to select “Not yet” if they are uncertain, and for the pediatrician to clarify the item during the preventive check-up. If the parent still cannot assess whether the behavior is present, it is recommended that the pediatrician treat the response as “not yet.” Even a parent’s inability to evaluate a particular behavior—whether due to lack of observation or other reasons—can provide valuable insight into the child’s psychosocial developmental context. Another potential limitation and recommendation for future improvement is to further explore the process of handling uncertain responses in the screening, ensuring that the tool remains effective and accurate in real-world practice. The consequence of this study lies primarily in drawing attention to the unidimensionality of the tool and highlighting the possibility of arranging items in the pediatric response sheet according to their difficulty level, rather than by subdomains such as motor skills, social behavior, language, etc., since these areas are interrelated and form a common construct. Additionally, we would consider incorporating more challenging items, especially for three-year-old children.

## Data Availability

The datasets presented in this article are not readily available because the data originate from the National Database and are not publicly available, but they can be shown upon request. Requests to access the datasets should be directed to lraczova@ukf.sk.
